# Inherited Variants in Wnt Pathway Genes Influence Outcomes of Prostate Cancer Patients Receiving Androgen Deprivation Therapy

**DOI:** 10.3390/ijms17121970

**Published:** 2016-11-26

**Authors:** Jiun-Hung Geng, Victor C. Lin, Chia-Cheng Yu, Chao-Yuan Huang, Hsin-Ling Yin, Ta-Yuan Chang, Te-Ling Lu, Shu-Pin Huang, Bo-Ying Bao

**Affiliations:** 1 Department of Urology, Kaohsiung Medical University Hospital, Kaohsiung 807, Taiwan; u9001090@hotmail.com; 2 Department of Urology, Kaohsiung Municipal Hsiao-Kang Hospital, Kaohsiung 812, Taiwan; 3 Department of Urology, E-Da Hospital, Kaohsiung 824, Taiwan; victorlin0098@yahoo.com.tw; 4 School of Medicine for International Students, I-Shou University, Kaohsiung 840, Taiwan; 5 Division of Urology, Department of Surgery, Kaohsiung Veterans General Hospital, Kaohsiung 813, Taiwan; ccyu@vghks.gov.tw; 6 Department of Urology, School of Medicine, National Yang-Ming University, Taipei 112, Taiwan; 7 Department of Pharmacy, Tajen University, Pingtung 907, Taiwan; 8 Department of Urology, National Taiwan University Hospital, College of Medicine, National Taiwan University, Taipei 100, Taiwan; cyhuang0909@ntu.edu.tw; 9 Department of Urology, National Taiwan University Hospital Hsin-Chu Branch, Hsinchu 300, Taiwan; 10 Department of Pathology, Kaohsiung Medical University Hospital, Kaohsiung 807, Taiwan; schoolyin@gmail.com; 11 Department of Pathology, Faculty of Medicine, College of Medicine, Kaohsiung Medical University, Kaohsiung 807, Taiwan; 12 Department of Occupational Safety and Health, China Medical University, Taichung 404, Taiwan; tychang@mail.cmu.edu.tw; 13 Department of Pharmacy, China Medical University, Taichung 404, Taiwan; lutl@mail.cmu.edu.tw; 14 Department of Urology, Faculty of Medicine, College of Medicine, Kaohsiung Medical University, Kaohsiung 807, Taiwan; 15 Graduate Institute of Medicine, College of Medicine, Kaohsiung Medical University, Kaohsiung 807, Taiwan; 16 Sex Hormone Research Center, China Medical University Hospital, Taichung 404, Taiwan; 17 Department of Nursing, Asia University, Taichung 413, Taiwan

**Keywords:** prostate cancer, androgen deprivation therapy, outcomes, genetic variation, Wnt pathway

## Abstract

Aberrant Wnt signaling has been associated with many types of cancer. However, the association of inherited Wnt pathway variants with clinical outcomes in prostate cancer patients receiving androgen deprivation therapy (ADT) has not been determined. Here, we comprehensively studied the contribution of common single nucleotide polymorphisms (SNPs) in Wnt pathway genes to the clinical outcomes of 465 advanced prostate cancer patients treated with ADT. Two SNPs, *adenomatous polyposis coli* (*APC*) rs2707765 and rs497844, were significantly (*p* ≤ 0.009 and *q* ≤ 0.043) associated with both prostate cancer progression and all-cause mortality, even after multivariate analyses and multiple testing correction. Patients with a greater number of favorable alleles had a longer time to disease progression and better overall survival during ADT (*p* for trend ≤ 0.003). Additional, cDNA array and in silico analyses of prostate cancer tissue suggested that rs2707765 affects *APC* expression, which in turn is correlated with tumor aggressiveness and patient prognosis. This study identifies the influence of inherited variants in the Wnt pathway on the efficacy of ADT and highlights a preclinical rationale for using *APC* as a prognostic marker in advanced prostate cancer.

## 1. Introduction

Prostate cancer is the most common type of cancer in men worldwide, with 1.1 million cases and 307,000 related deaths in 2012. Most cases of prostate cancer are diagnosed and treated while the disease is localized. However, 10%–20% present with advanced-stage or metastatic prostate cancer, and others develop disseminated disease after definitive treatment [1]. Once an advanced-stage or metastatic prostate cancer is diagnosed, androgen deprivation therapy (ADT), which can be accomplished with either bilateral surgical or medical castration, is the standard systemic therapy [2,3]. Although the initial response rate of prostate cancer to ADT has been reported to be as high as 80%, ADT is not curative for prostate cancer, and many patients receiving ADT progress to castration-resistant prostate cancer (CRPC) within 18–30 months [4]. Once CRPC develops, a patient’s life expectancy is approximately 16–18 months [5]. Several parameters, including the prostate-specific antigen (PSA) doubling time, PSA nadir, PSA level at ADT initiation, stage and Gleason score, have been reported as useful prognostic predictors of disease progression or survival in patients receiving ADT. However, the predictive ability of these parameters remains limited and might be improved by incorporating information on genetic variants, which may help to estimate disease progression and identify novel therapeutic targets.

The Wnt pathway is an evolutionarily-conserved signal transduction pathway that governs embryonic growth by directing processes, such as cell fate decisions, proliferation, neural patterning, polarity, migration and apoptosis [6,7]. The accumulated evidence has demonstrated that Wnt pathway aberrations are frequently associated with tumor development and the progression of many types of cancer, including colorectal cancer, hepatocellular cancer, breast cancer [8,9,10,11,12,13,14,15] and prostate cancer [16,17,18,19,20]. The best-known example, familial adenomatous polyposis, is an autosomal, dominantly inherited disease characterized by the development of polyps in the colon and rectum. It is most frequently caused by a mutation of the gene *adenomatous polyposis coli* (*APC*) [21,22]. In addition, mutations in β-catenin (*CTNNB1*) and *APC* have been identified in sporadic colorectal cancers and various other tumor types [15]. Although oncogenic mutations in Wnt pathway genes are rare in prostate cancer, increased expression levels of Wnt family proteins have been observed in clinical prostate cancer samples [18,23]. Additionally, the treatment of LNCaP human prostate cancer cells with conditioned medium containing the growth factor Wnt3a significantly enhanced cell growth in the absence of androgens, demonstrating that the Wnt pathway may represent a novel mechanism contributing to prostate cancer progression [24]. Therefore, it is reasonable to hypothesize that genetic variants in the Wnt signaling pathway could influence prostate cancer progression and might also contribute to the development of CRPC. In this study, we comprehensively evaluated the prognostic significance of 17 tagged single nucleotide polymorphisms (SNPs) in three key genes related to the Wnt pathway, *WNT1*, *APC* and *CTNNB1*, with regards to disease progression and all-cause mortality (ACM) in a cohort of prostate cancer patients treated with ADT.

## 2. Results

The demographic and clinicopathologic characteristics of the study participants are summarized in Table S1. With a median follow-up time of 92 months, 429 (92.3%) prostate cancer patients experienced disease progression after ADT. A total of 143 (30.8%) patients died at a median follow-up of 65 months. The clinical stage, Gleason score, PSA at ADT initiation, PSA nadir, time to PSA nadir and treatment modalities were associated with both disease progression and ACM (*p* ≤ 0.004). The age at diagnosis was only correlated with the disease progression (*p* < 0.001).

**Figure 1:**
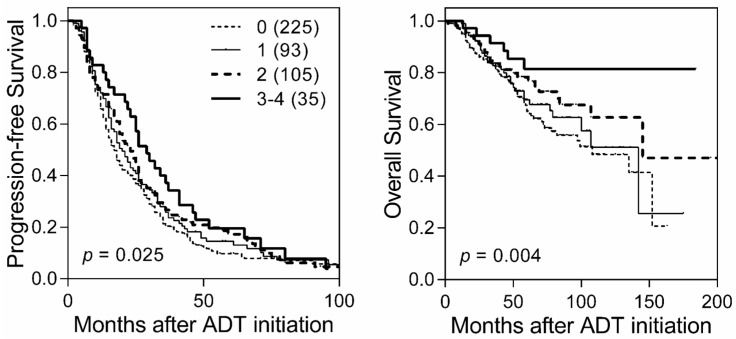
Kaplan-Meier curves of time to progression (**left** panel) and all-cause mortality (ACM) (**right** panel) during androgen deprivation therapy (ADT) for patients with 0, 1, 2 or 3–4 protective alleles at *APC* rs2707765 and rs497844. The protective alleles refer to C in rs2707765 and T in rs497844. The more protective alleles a prostate cancer patient carries, the longer their time to progression and ACM. Numbers in parentheses indicate the number of patients.

Seventeen tagging SNPs in three core Wnt pathway genes, namely *CTNNB1*, *APC* and *WNT1*, were analyzed in this study. Details of the SNPs and their associations with disease progression and ACM during ADT are shown in Table S2. Seven *APC* SNPs were associated with disease progression or ACM with a nominal *p* < 0.05, according to a multivariate Cox model adjusted for age, clinical stage, Gleason score, PSA at ADT initiation, PSA nadir, time to PSA nadir and treatment modality. After adjusting for the false discovery rate (FDR) at the <0.05 level, four SNPs, namely *APC* rs3846716, rs2707765, rs41115 and rs497844, remained significantly associated with time to progression (*q* ≤ 0.012; Table 1). Intriguingly, *APC* rs2707765 and rs497844 correlated significantly with a decreased risk of both disease progression and ACM (*q* ≤ 0.043; Table 2). A gene-dosage effect on disease progression and ACM was found when two genetic loci of interest were analyzed in combination, with the hazards ratio decreasing as the number of protective alleles increased (*p* for trend ≤ 0.003; Table 3 and Figure 1).

**Figure 2:**
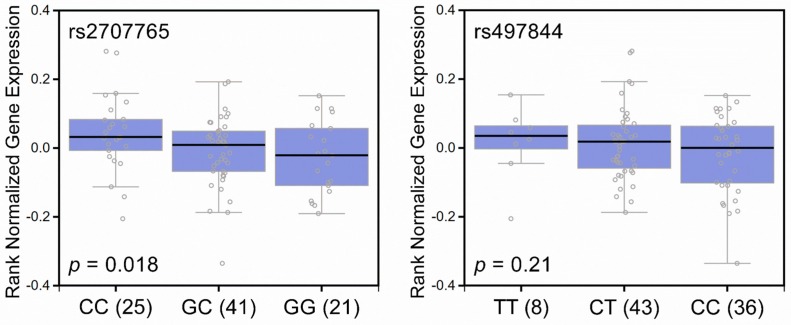
Correlation of rs2707765 and rs497844 genotypes and *APC* gene expression in prostate tissues. Boxplots represent *APC* mRNA expression according to the rs2707765 (**left** panel) and rs497844 (**right** panel) genotypes in 87 human prostate tissues (Genotype-Tissue Expression (GTEx) dataset). There is a trend toward increased *APC* mRNA expression in the prostate tissues of rs2707765 protective allele C carriers and rs497844 protective allele T carriers. The numbers in parentheses indicate the number of cases.

As a preliminary assessment of the putative functional roles of these SNPs, we investigated whether rs2707765 and rs497844 were associated with the differential expression of *APC*. The Genotype-Tissue Expression (GTEx) database revealed a significant trend toward increased *APC* mRNA expression in the prostate tissues of rs2707765 protective allele (C) carriers (*p* = 0.018; Figure 2, left) and rs497844 protective allele (T) carriers (*p* = 0.21; Figure 2, right). We used HaploReg to annotate these risk-associated SNPs and discover their potential causal link with the disease. rs2707765, rs497844 and several SNPs in strong linkage disequilibrium with them (*r*^2^ > 0.8) (e.g., rs2431514 and rs2464803 for rs2707765 and rs712671 and rs455469 for rs497844) are situated within a locus showing promoter and enhancer histone marks, DNase hypersensitivity peaks and transcription factor binding in several cell lines (Tables S3 and S4). Together, rs2707765 and rs497844 might affect transcription factor binding, increase *APC* mRNA expression and reduce the aggressiveness of prostate cancer.

**Figure 3:**
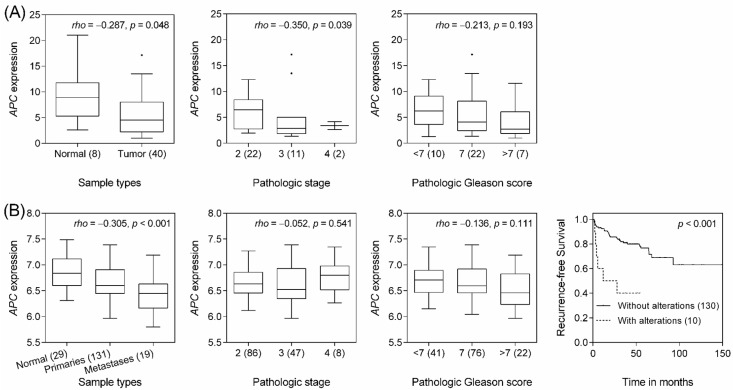
Correlation of *APC* mRNA expression with prostate cancer progression. (**A**) *APC* mRNA expression in 40 prostate cancers and eight normal human prostate tissue specimens, as determined by qRT-PCR, indicates that *APC* is downregulated in the cancer tissues, especially in those from patients with more advanced stages of cancer; (**B**) The associations between *APC* expression and prostate cancer aggressiveness were analyzed using an independent set of Memorial Sloan-Kettering Cancer Center Prostate Oncogenome data. Primary and metastatic prostate cancers display significantly lower *APC* mRNA expression. *rho*, Spearman’s rank correlation coefficient. Data points beyond the quartiles are outliers. Kaplan-Meier curves of recurrence-free survival according to the alterations in *APC* are shown. Patients were dichotomized with or without *APC* homozygous deletion, mutation and mRNA downregulation. Numbers in parentheses indicate the number of patients.

To determine whether *APC* expression is linked to prostate cancer progression, we performed a quantitative real-time polymerase chain reaction (qRT-PCR) analysis of *APC* transcripts using a prostate cancer complementary DNA (cDNA) array containing 48 tissue samples. A significant downregulation of *APC* expression was observed in cancer and more advanced stage cancer samples (*p* ≤ 0.048; Figure 3A). To further confirm the relationship between *APC* expression and prostate cancer aggressiveness, we performed an in silico evaluation using publicly-available Memorial Sloan-Kettering Cancer Center Prostate Oncogenome datasets [25]. Our analysis revealed that more clinically-advanced prostate cancers, such as those with metastasis, had significantly lower levels of *APC* expression (*p* < 0.001; Figure 3B). Follow-up of this cohort established that *APC* homozygous deletion, mutation and mRNA downregulation were strongly associated with a worse recurrence-free survival (*p* < 0.001, Figure 3B).

## 3. Discussion

In this hypothesis-driven association study, we identified two SNPs in *APC*, namely rs2707765 and rs497844, as being associated with the efficacy of ADT. Notably, the relationships of these SNPs with disease progression and ACM persisted despite controlling for known prognostic factors and multiple comparison testing. This suggests that the patient genotype adds information beyond conventional factors. Moreover, rs2707765 affects *APC* expression, and a lower *APC* expression correlated with more aggressive cancer and a worse clinical outcome. These results support the connection between the Wnt pathway and prostate cancer progression.

APC is a multifunctional protein involved in a variety of cellular processes. Its best-known role is as a negative regulator of the Wnt pathway, but recent evidence also suggests that it has a role in regulating microtubule dynamics in mitosis. APC was found to associate with the plus-ends of microtubules that interact with kinetochores and to be required for the spindle checkpoint to prevent chromosome missegregation [26]. Many tumors exhibit chromosomal instability, which is probably caused by chromosome missegregation [27]. *APC* mutant mouse embryonic stem cells were reported to have multipolar spindles, indicating that *APC* mutations could lead to chromosomal instability through defective mitosis [28,29]. Therefore, it was postulated that mutations in *APC* lead to chromosomal instability and induce aberrant Wnt signaling, thus contributing to tumor progression. While *APC* is highly relevant with respect to chromosomal aberrations in human colorectal cancers [21,22], mutations in *APC* are rare in human prostate cancer [30]. Many studies have focused on *APC* hypermethylation, which might cause *APC* downregulation [31,32] and has been associated with clinicopathological features of tumor aggressiveness. In a systematic review and meta-analysis of studies on tissue samples, *APC* promoter hypermethylation was found to correlate with more advanced stages of prostate cancer [33]. Functional annotations derived from the Encyclopedia of DNA Elements (ENCODE) and Roadmap Epigenomics data indicate that rs2707765 and rs497844, as well as their linked SNPs, coincide with open chromatin regions that likely correspond to promoters or enhancers of *APC* (Tables S3 and S4). In addition, rs2707765 and rs497844 are predicted to influence the binding of several transcription factors, such as fetal Alz-50 reactive clone 1 (FAC1), activating transcription factor 3 (ATF3) and the E2 factor (E2F) family of transcription factors. These data were consistent with our findings that the protective alleles, rs2707765 C and rs497844 T, tend to have a higher expression of *APC* and a better response to therapy. Interestingly, our previous study also demonstrated a potential prognostic role of *APC* rs3846716 (although it did not reach the predefined significance threshold of *q* < 0.05 in ACM) with regards to post-radical prostatectomy recurrence for localized prostate cancer [34]. Taken together, these results highlight the importance of *APC* throughout all stages of cancer treatment.

The present study has several strengths. This is the first study to have assessed whether genetic variants in the Wnt pathway influence clinical outcomes for advanced prostate cancer patients receiving ADT. Using a cDNA array and after validation with a published dataset, we have also presented additional evidence for a role of *APC* in prostate cancer, namely that downregulated APC gene expression was associated with more aggressive cancer and a poor prognosis. The haplotype-tagging SNP approach ensured the exhaustive and detailed coverage of susceptibility alleles across the whole *APC* gene region. Moreover, complete clinical information and follow-up data allowed us to control for potential confounding factors. However, our study population comprised homogeneous Han Chinese individuals, and therefore, the findings might be less applicable to other ethnic groups. There are many important Wnt pathway genes that have not been investigated in the present study. Thus, further validation in larger interethnic cohorts and additional functional studies will be necessary to elucidate the underlying biological mechanisms.

## 4. Materials and Methods

### 4.1. Patient Selection and Data Collection

Our study population included prostate cancer patients treated from 1995–2009 at three medical centers in Taiwan: Kaohsiung Medical University Hospital (Kaohsiung, Taiwan), Kaohsiung Veterans General Hospital (Kaohsiung, Taiwan) and National Taiwan University Hospital (Taipei, Taiwan), as described previously [35]. The clinical characteristics at diagnosis were compiled from medical records, and patients were followed-up prospectively to evaluate whether the tested genetic variations could represent prognostic factors of clinical outcomes during ADT. After excluding patients with inadequate clinicopathological characteristics or follow-up periods, 465 patients remained in the cohort. Written informed consent was obtained from all participants, and the study was approved by the Institutional Review Board of Kaohsiung Medical University Hospital (#KMUHIRB-2013132; 21 January 2014; Kaohsiung, Taiwan).

Treatment outcomes, disease progression and ACM were measured and updated recently during a prospective follow-up in 2014. The PSA nadir was defined as the lowest PSA value achieved at any time during ADT treatment [36,37]. The time to PSA nadir was defined as the duration from the initiation of ADT to the PSA nadir [38]. Disease progression was defined as the presence of at least two consecutive increases in the PSA value more than one week apart that exceeded the PSA nadir [39]. The use of secondary hormone treatment for increasing PSA was also considered to be a progression event. The time to progression was defined as the duration from the start of ADT until disease progression. In this study, patients underwent regular monthly follow-up evaluations and PSA testing at three-month intervals. Causes of death were determined from the official cause of death registry provided by the Department of Health, Executive Yuan, Taipei, Taiwan. ACM was defined as the interval from ADT initiation to death from any cause.

### 4.2. Single Nucleotide Polymorphism (SNP) Selection and Genotyping

We used a tagging SNP approach to select genetic variants for the investigation of the genetic variability in three core genes of the Wnt pathway, namely *WNT1*, *APC* and *CTNNB1*. Eighteen tagged SNPs were selected from Han Chinese in Beijing HapMap data using Tagger with *r*^2^ ≥ 0.8 and a minor-allele frequency ≥ 0.2 [40,41]. Genomic DNA was extracted from the peripheral blood using QIAamp DNA Blood Mini Kit (Qiagen, Venlo, The Netherlands) following the manufacturer’s instruction, and stored at −80 °C. Genotyping was performed using iPLEX^®^ matrix-assisted laser desorption/ionization time-of-flight mass-spectrometry technology (Agena Bioscience, Hamburg, Germany) at the National Center for Genome Medicine, Academia Sinica, Taipei, Taiwan, as described previously [42]. The average genotype call rate for genotyped SNPs was 99.7%, and the average concordance rate was 100% among five duplicated control samples. One SNP, rs1798802, significantly deviated from the Hardy-Weinberg equilibrium (*p* < 0.05) and was removed, leaving 17 SNPs for further statistical analysis.

### 4.3. Human Tissue cDNA Array and TaqMan^®^ qRT-PCR Analysis

The expression levels of *APC* and *β-actin* (*ACTB*) were measured using the TissueScan™ human prostate cancer cDNA array II, including 8 normal and 40 prostate cancer samples (OriGene Technologies, Rockville, MD, USA). qRT-PCR was performed using prevalidated TaqMan^®^ gene expression assays (Applied Biosystems, Foster City, CA, USA) for *APC* (Hs01568269_m1) and *ACTB* (Hs01060665_g1), on a 7500 real-time PCR system (Applied Biosystems, Foster City, CA, USA) according to the manufacturer’s instructions. The quantification of each sample was normalized using the house-keeping gene *ACTB*.

### 4.4. Statistical Analysis

The clinical characteristics of the study population were summarized as numbers and percentages of patients or median values with interquartile ranges. Associations of clinical characteristics with disease progression and ACM were estimated using the log-rank test or Cox regression analysis. The median follow-up time and 95% confidence intervals of disease progression and ACM were assessed using the reverse Kaplan-Meier method. Associations of genotyped SNPs with the time to progression and ACM were assessed using multivariate Cox models adjusted for known prognostic factors, including age, clinical stage, Gleason score, PSA at ADT initiation, PSA nadir, time to PSA nadir and treatment modality. For each SNP, three genetic models (dominant, recessive and additive) of inheritance were assessed. The model with the strongest likelihood was considered to be the best model for each SNP. Only additive and dominant models were assessed if the variant homozygotes were observed in <0.05 of the study population. While testing the selected 17 SNPs, the FDR was calculated to determine the degree to which the tests for association were prone to yielding false positive results, using the *q*-values method [43]. *q*-Values and two-sided *p*-values < 0.05 were considered to be statistically significant. SPSS software, Version 22.0.0 (IBM, Armonk, NY, USA), was used for the statistical analyses.

### 4.5. Bioinformatics Analysis

HaploReg Version 4.1 [44] was used to assess the evidence of regulatory potential for the associated SNPs. We used the GTEx data to examine the correlations between SNPs and gene expression levels in human prostate tissues [45]. To further validate our findings, the association between *APC* expression and prostate cancer aggressiveness was analyzed using data from the Memorial Sloan-Kettering Cancer Center Prostate Oncogenome [25].

## 5. Conclusions

We comprehensively evaluated haplotype-tagging SNPs in core genes of the Wnt pathway and provided the first evidence of an association between *APC* gene variants and outcomes in advanced prostate cancer patients. Our findings might influence personalized prostate cancer management by identifying patients who would not benefit from ADT.

## Figures and Tables

**Table 1:** Association between htSNPs in *APC* and disease progression in prostate cancer patients treated with ADT.

SNP	Location	Allele	Event ^a^	No Event ^a^	Best Model	HR (95% CI) ^b^	*p* ^b^	*q*
rs3846716	5′ upstream	G>A	295/117/12	22/11/2	Dominant	0.67 (0.53–0.84)	<0.001	**0.009**
rs2289485	Intron	T>G	371/54/2	30/5/1	Additive	0.74 (0.56–0.99)	0.040	0.093
rs2707765	Intron	G>C	210/176/38	16/15/4	Additive	0.78 (0.67–0.91)	0.002	**0.009**
rs2431238	Intron	C>T	343/76/2	30/4/1	Additive	0.77 (0.60–0.99)	0.044	0.093
rs41115	Thr1493Thr	T>C	300/114/14	25/9/2	Dominant	0.71 (0.57–0.89)	0.003	**0.012**
rs497844	3′ downstream	C>T	301/107/15	24/10/1	Dominant	0.68 (0.54–0.85)	0.001	**0.009**

Abbreviations: htSNP, haplotype tagging single nucleotide polymorphism; *APC*, *adenomatous polyposis coli*; ADT, androgen deprivation therapy; HR, hazards ratio; CI, confidence interval; *p*, *p*-value; *q*, *q*-value. ^a^ The number represents major allele homozygotes, heterozygotes and minor allele homozygotes, respectively; ^b^ Multivariate Cox models adjusted for age, clinical stage, Gleason score, prostate-specific antigen (PSA) at ADT initiation, PSA nadir, time to PSA nadir and treatment modality. The major allele homozygotes were considered as the reference group, with a fixed HR = 1.00. *p* < 0.05 is in boldface.

**Table 2:** Association between htSNPs in *APC* and ACM in prostate cancer patients treated with ADT.

SNP	Location	Allele	Event ^a^	No Event ^a^	Best Model	HR (95% CI) ^b^	*p* ^b^	*q*
rs3846716	5′ upstream	G>A	106/32/2	211/96/12	Additive	0.65 (0.44–0.95)	0.026	0.055
rs2289485	Intron	T>G	129/12/0	272/47/3	Additive	0.46 (0.25–0.86)	0.014	0.054
rs2707765	Intron	G>C	77/54/9	149/137/33	Additive	0.68 (0.51–0.91)	0.009	**0.043**
rs17134945	Intron	A>G	130/12/0	276/43/3	Additive	0.52 (0.28–0.95)	0.033	0.057
rs41115	Thr1493Thr	T>C	108/31/3	217/92/13	Dominant	0.62 (0.41–0.95)	0.026	0.055
rs497844	3′ downstream	C>T	110/27/3	215/90/13	Dominant	0.51 (0.33–0.80)	0.003	**0.043**

Abbreviations: ACM, all-cause mortality. ^a^ The number represents major allele homozygotes, heterozygotes and minor allele homozygotes, respectively; ^b^ Multivariate Cox models adjusted for age, clinical stage, Gleason score, PSA at ADT initiation, PSA nadir, time to PSA nadir and treatment modality. The major allele homozygotes were considered as the reference group, with a fixed HR = 1.00. *p* < 0.05 is in boldface.

**Table 3:** Cumulative effect of predictive markers on disease progression and ACM in prostate cancer patients treated with ADT.

No. of Protective Alleles ^a^	Overall Patients, *n*	Disease Progression				ACM			
		Events, *n*	Median, *mo*	HR (95% CI) ^b^	*p* ^b^	Events, *n*	Median, *mo*	HR (95% CI) ^b^	*p* ^b^
0	225	109	17	1.00	-	77	108	1.00	-
1	93	86	20	0.91 (0.70–1.18)	0.467	30	142	0.76 (0.48–1.20)	0.240
2	105	96	23	0.72 (0.55–0.93)	**0.012**	27	145	0.60 (0.37–0.97)	**0.037**
3–4	35	32	29	0.56 (0.38–0.83)	**0.004**	6	NR ^c^	0.36 (0.16–0.84)	**0.018**
Trend	-	-	-	0.84 (0.76–0.93)	**<0.001**	-	-	0.75 (0.62–0.90)	**0.003**

Abbreviations: mo, month. ^a^ Protective alleles refer to C in rs2707765 and T in rs497844; ^b^ Multivariate Cox models adjusted for age, clinical stage, Gleason score, PSA at ADT initiation, PSA nadir, time to PSA nadir and treatment modality; ^c^ NR means the median time-to-event has not been reached. *p* < 0.05 is in boldface.

## Supplementary Materials

Supplementary materials can be found at www.mdpi.com/1422-0067/17/12/1970/s1.
