# Supplementary material for: Inherited Variants in Wnt Pathway Genes Influence Outcomes of Prostate Cancer Patients Receiving Androgen Deprivation Therapy
Source: Int J Mol Sci. 2016 Nov 26;17(12):1970. doi: 10.3390/ijms17121970 (PMC5187770; doi:10.3390/ijms17121970)
Supplement: Supplementary file 1 [file ijms-17-01970-s001.pdf]

Supplementary Material: Inherited Variants in Wnt Pathway Genes Influence Outcomes of Prostate Cancer Patients Receiving Androgen Deprivation Therapy

Jiun-Hung Geng, Victor C. Lin, Chia-Cheng Yu, Chao-Yuan Huang, Hsin-Ling Yin, Ta-Yuan Chang, Te-Ling Lu, Shu-Pin Huang and Bo-Ying Bao

Table S1. Clinical characteristics of the study population.

Characteristic	*n* (%)	Progression	ACM
		p [a]	*p* [a]
Patients, *n*	465		
Age at diagnosis, *y*			
Median (IQR)	73 (67–79)	<0.001	0.182
Clinical stage at diagnosis			
M0	269 (58.1)	<0.001	<0.001
M1	194 (41.9)		
Gleason score at diagnosis			
≤7	284 (62.7)	<0.001	<0.001
>7	169 (37.3)		
PSA at ADT initiation, ng/mL			
Median (IQR)	40.8 (12.9–144)	<0.001	<0.001
PSA nadir, ng/mL			
Median (IQR)	0.16 (0.01–1.21)	<0.001	<0.001
Time to PSA nadir, *mo*			
Median (IQR)	11 (5–21)	<0.001	<0.001
Treatment modality			
ADT as primary treatment	254 (54.7)	0.004	<0.001
ADT for post RP PSA failure	35 (7.5)		
ADT for post RT PSA failure	12 (2.6)		
Neoadjuvant/adjuvant ADT with RT	121 (26.1)		
Others	42 (9.1)		
Disease progression	429 (92.3)		
Median follow-up time [b], *mo* (95% CI)	92 (83–101)		
ACM	143 (30.8)		
Median follow-up time [b], *mo* (95% CI)	65 (60–70)		

Abbreviations: ACM, all-cause mortality; *y*, year; IQR, interquartile range; M0, no distant metastasis; M1, metastasis to distant organs; PSA, prostate-specific antigen; *mo*, month; ADT, androgen deprivation therapy; RP, radical prostatectomy; RT: radiotherapy; CI, confidence interval. [a] The *p*-value was calculated by log-rank test or Cox regression; [b] Median follow-up time and 95% CIs were estimated with the reverse Kaplan-Meier method.

Table S2. Genotyped SNPs and the *p*-values of their association with time to progression and ACM during ADT.

Gene	SNP ID	Chromosome	Position	Progression			ACM		
				Additive	Dominant	Recessive	Additive	Dominant	Recessive
CTNNB1	rs4016435	3	41225133	0.238	0.217	-	0.264	0.242	-
CTNNB1	rs11564459	3	41251411	0.946	0.946	-	0.611	0.611	-
CTNNB1	rs11564465	3	41252044	0.292	0.257	-	0.855	0.733	-
CTNNB1	rs4135385	3	41254444	0.635	0.798	0.249	0.709	0.377	0.700
CTNNB1	rs11564475	3	41255037	0.085	0.166	-	0.501	0.534	-
CTNNB1	rs2293303	3	41255831	0.198	0.200	-	0.464	0.584	-
APC	rs3846716	5	112087493	**0.002**	**<0.001**	-	**0.026**	**0.029**	-
APC	rs2289485	5	112144628	**0.040**	0.054	-	**0.014**	**0.017**	-
APC	rs2707765	5	112150431	**0.002**	**0.006**	**0.029**	**0.009**	**0.009**	0.158
APC	rs2431238	5	112152268	**0.044**	**0.049**	-	0.418	0.455	-
APC	rs17134945	5	112167921	0.083	0.116	-	**0.033**	**0.041**	-
APC	rs2707761	5	112177826	0.095	0.095	-	0.240	0.162	-
APC	rs41115	5	112203669	**0.005**	**0.003**	-	**0.026**	**0.026**	-
APC	rs497844	5	112213390	**0.002**	**0.001**	-	**0.005**	**0.003**	-
WNT1	rs4018511	12	47641303	0.965	0.712	0.549	0.931	0.938	0.724
WNT1	rs833841	12	47652105	0.652	0.807	0.594	0.361	0.300	0.687
WNT1	rs10783298	12	47671224	0.620	0.437	0.733	0.704	0.569	0.819

Abbreviations: SNP, single nucleotide polymorphism. The *p*-values were calculated using the multivariate Cox models adjusted for age, clinical stage, Gleason score, PSA at ADT initiation, PSA nadir, time to PSA nadir and treatment modality. $p < 0.05$ is in boldface.

Table S3. Regulatory annotation of variants linked with rs2707765.

Chromosome	Position	LD (r^2)	SNP ID	Reference Allele	Alternate Allele	ASN Frequency	Variant Type	Promoter Histone Marks	Enhancer Histone Marks	DNAse	Proteins Bound	Motifs Changed
5	112753585	0.84	rs11241183	T	C	0.67	intronic		ESDR, BRN			
5	112759443	0.82	rs6879057	C	T	0.67	intronic					BCL, ERalpha-a, Ets, Irf, SP1, ZBRK1
5	112760078	0.83	rs4705624	T	A	0.67	intronic					Crx, Evi-1, GATA, LUN-1
5	112760929	0.83	rs2901844	C	T	0.67	intronic					AP-1, Ik-1, Maf
5	112761537	0.83	rs28578275	G	A	0.67	intronic		FAT, STRM, MUS, LNG, SKIN			Hoxb3, RFX5
5	112767983	0.83	rs2431239	G	A	0.67	intronic					Maf
5	112768266	0.83	rs2546117	C	G	0.67	intronic					Hoxb3, RAR, RFX5
5	112768600	0.83	rs35414976	A	G	0.67	intronic					Cdx2, GATA, Hlx1, Hoxa10, Hoxa9, Hoxb8, Hoxd10, Pou3f2
5	112768600	0.82	rs140904242	TG	T	0.66	intronic					Hoxa9, Hoxb9
5	112769525	0.82	rs142579546	TA	T	0.66	intronic					Foxd3, Hoxa9, Isl2
5	112772029	0.87	rs2439589	C	T	0.67	intronic	SKIN	FAT, STRM, MUS, SKIN, GI, ADRL, BRN, LNG, BONE			GR, NRSF
5	112776169	0.86	rs458906	G	C	0.66	intronic		FAT, MUS, SKIN, LNG			Foxp1, Pou2f2
5	112777830	0.82	rs467033	A	T	0.68	intronic		SKIN			Hand1
5	112781076	0.93	rs2289484	A	G	0.68	intronic					Cart1, Foxa
5	112783259	0.88	rs2431512	C	T	0.67	intronic					Arid5b, Pou5f1, Spz1
5	112786835	1	**rs2707765**	G	C	0.7	intronic		LNG, SKIN	ESDR, LNG		FAC1
5	112786906	0.81	rs34244415	GAGTC	G	0.67	intronic		LNG, BRST, MUS, SKIN, PLCNT	ESDR		AP-1, GATA, HMGN3, Irf, KAP1, TCF11::MafG
5	112795591	0.94	rs2431514	A	G	0.7	intronic	LNG, BLD	ESC, IPSC, BLD, VAS	ESC, ESDR, IPSC, IPSC, BLD	CEBPB, TAL1, GATA1	GATA, Mrg, PPAR, Pbx3, Tgif1, VDR
5	112796090	0.94	rs2464803	A	G	0.7	intronic	ESC, IPSC, LNG, BLD	ESC, ESDR, IPSC, STRM, BLD, MUS, PANC, LIV, VAS	ESC, BLD, VAS, BLD	GATA2, TAL1	AIRE, DEC, Foxa, Foxo, Irf, Sox, Zfp105
5	112799393	0.94	rs518013	G	A	0.7	intronic		IPSC, BRN, LNG			Pax-4, RREB-1, RXRA
5	112800040	0.94	rs2251913	A	G	0.7	intronic		SKIN, BRN			Foxa

Table S3. *Cont.*

Chromosome	Position	LD (r^2)	SNP ID	Reference Allele	Alternate Allele	ASN Frequency	Variant Type	Promoter Histone Marks	Enhancer Histone Marks	DNAse	Proteins Bound	Motifs Changed
5	112800507	0.94	rs511906	A	G	0.7	intronic		BRN			Arid3a, CEBPA, Cdx2, Dlx3, HNF1, Hoxa10, Hoxc9, Hoxd10, Lhx3, Nkx6-1, Pax-6, Pdx1, Pou2f2, Pou3f2, Pou5f1, Prrx2, SIX5, STAT
5	112804048	0.93	rs2545158	A	G	0.7	intronic			MUS		
5	112808326	0.91	rs2431241	A	G	0.71	intronic					Cart1, HNF4, Pou2f2
5	112808747	0.86	rs1914	A	T	0.7	intronic					CTCF, Maf, Rad21
5	112809564	0.9	rs2546106	C	A	0.69	intronic					Mxi1, SREBP
5	112810500	0.86	rs390092	T	G	0.69	intronic		PANC			Myb, YY1
5	112823773	0.85	rs2546107	A	G	0.7	intronic		BLD			Bcl6b, PTF1-beta, STAT
5	112825772	0.89	rs2546108	C	A	0.68	intronic		ESC, IPSC			PU.1, Pou5f1
5	112826716	0.83	rs2546110	A	G	0.69	intronic		ESDR, ESC			Sox
5	112827157	0.86	rs2229992	T	C	0.69	synonymous					
5	112829165	0.87	rs351772	G	A	0.68	intronic					
5	112831165	0.84	rs2253987	G	A	0.68	intronic					ZBTB33, Zbtb3
5	112831890	0.85	rs548710	T	C	0.69	intronic					
5	112837233	0.84	rs1966476	T	C	0.69	intronic					DMRT7, Homez, SIX5

Abbreviations: LD, linkage disequilibrium; ASN, Asian; DNAse; DNase hypersensitivity. The risk SNP identified in this study is in boldface.

Table S4. Regulatory annotation of variants linked with rs497844.

Chromosome	Position	LD (r^2)	SNP ID	Reference Allele	Alternate Allele	ASN Frequency	Variant Type	Promoter Histone Marks	Enhancer Histone Marks	DNAse	Proteins Bound	Motifs Changed
5	112793497	0.81	rs11291630	AC	A	0.81	intronic		ESC, ESDR, IPSC, STRM, PLCNT			Foxo, Hoxb13, Ik-2, NF-AT, NF-AT1, RREB-1
5	112803191	0.83	rs2952615	G	C	0.81	intronic					Foxp3, Nrf-2
5	112815854	0.83	rs12656359	G	T	0.81	intronic					GR, HNF4, Roaz
5	112816952	0.8	rs2431240	C	T	0.8	intronic					CHOP::CEBPalpha, ERalpha-a, Myc, Rad21, SP1, Zfp740
5	112817422	0.85	rs2545165	C	T	0.81	intronic					Hltf, NF-kappaB, SRF
5	112817968	0.85	rs2545164	C	T	0.81	intronic					Foxc1
5	112818454	0.85	rs57559075	ATAT	A	0.81	intronic					Arid5a, Dbx2, Gsc, HNF1, Hoxc9, Hoxd10, Nkx6-1, Pou2f2, Pou3f2, Pou3f4, Pou4f3
5	112818606	0.85	rs464708	T	G	0.81	intronic					Eomes
5	112822734	0.82	rs2545162	G	A	0.81	intronic		ESDR, STRM	SKIN		Cart1, Nkx3
5	112826601	0.84	rs139232022	AC	A	0.81	intronic		ESDR, ESC, IPSC			FAC1, Foxd3, Foxj1, Foxk1, Foxo, Foxp1, Irf, SIX5, Sox
5	112828541	0.84	rs187075	C	T	0.81	intronic					Egr-1, Foxa, LUN-1, SETDB1, STAT
5	112828864	0.85	rs351771	G	A	0.81	synonymous					CHOP::CEBPalpha
5	112832124	0.85	rs569940	C	T	0.81	intronic					Barhl1, Barx2, CHX10, Dbx1, En-1, Esx1, FXR, Gbx1, Gbx2, Hlx1, Hoxb7, Hoxb8, Hoxc6, Hoxd8, Lhx4, Mef2, Msx-1, Msx2, Ncx, Nkx6-1, Pax7, Pdx1, Pou3f2, Pou3f4, Pou4f3, Pou6f1
5	112832244	0.87	rs2909958	C	G	0.81	intronic					Myb
5	112832368	0.85	rs2909786	A	G	0.81	intronic					Pbx-1, Pbx3
5	112832433	0.85	rs2909787	G	C	0.81	intronic					BCL, Brachyury, DMRT4, GATA, HMGN3, TCF11::MafG

Table S4. *Cont.*

Chromosome	Position	LD (r^2)	SNP ID	Reference Allele	Alternate Allele	ASN Frequency	Variant Type	Promoter Histone Marks	Enhancer Histone Marks	DNAse	Proteins Bound	Motifs Changed
5	112833439	0.85	rs383256	T	C	0.81	intronic					Glis2, HNF4, Ik-1, RXRA, ZBTB7A
5	112836522	0.88	rs411356	G	A	0.81	intronic					Maf, PLZF, Pou2f2
5	112837089	0.88	rs1966477	G	T	0.81	intronic					
5	112840073	0.85	rs41115	G	A	0.81	synonymous					Nkx2
5	112840628	0.85	rs42427	G	A	0.81	synonymous					MAZR, Pax-5, RXRA, SP1, UF1H3BETA, ZNF263
5	112840862	0.85	rs866006	T	G	0.81	synonymous					
5	112841474	0.93	rs465899	G	A	0.81	synonymous					HP1-site-factor, Irf, STAT
5	112845879	0.92	rs397768	G	A	0.81	3'-UTR		ESDR			MAZ
5	112846535	0.95	rs433429	A	T	0.82			ESC, ESDR, IPSC, BRN, SKIN, HRT			GATA, NRSF
5	112846667	0.95	rs11285673	GT	G	0.82			ESC, ESDR, IPSC, BRN, SKIN, HRT	ESDR, ESC		AP-1, RBP-Jkappa
5	112846682	0.95	rs386830	G	A	0.82			ESC, ESDR, IPSC, BRN, SKIN, HRT	ESDR, ESC		AP-1, Nkx2, Pax-2, RXRA
5	112849374	0.81	rs11377569	A	AA, AAT	0.78						
5	112849696	0.98	rs565453	A	C	0.81						Alx4, Nkx3, Sox, TATA
5	112849754	1	rs565603	A	G	0.81			ESDR			TCF11::MafG
5	112849794	1	**rs497844**	A	G	0.81			ESDR			ATF3, E2F, Jundm2
5	112850367	0.96	rs481789	C	A	0.81						PLZF, Pdx1
5	112858335	0.95	rs580237	A	C	0.82						Hltf, Pou5f1
5	112858996	0.94	rs429427	A	G	0.81						YY1

Table S4. *Cont.*

Chromosome	Position	LD (r^2)	SNP ID	Reference Allele	Alternate Allele	ASN Frequency	Variant Type	Promoter Histone Marks	Enhancer Histone Marks	DNAse	Proteins Bound	Motifs Changed
5	112861940	0.95	rs712668	T	G	0.81	intronic	ESC, ESDR, LNG, IPSC, FAT, STRM, BRST, BLD, MUS, BRN, SKIN, VAS, LIV, GI, ADRL, HRT, KID, PANC, PLCNT, THYM, OVRY, SPLN, CRVX, BONE				CEBPD, PRDM1, RXRA, p300
5	112862289	0.95	rs431287	A	T	0.81	intronic	ESC, ESDR, LNG, IPSC, FAT, STRM, BRST, BLD, MUS, BRN, SKIN, VAS, LIV, GI, ADRL, HRT, KID, PANC, PLCNT, THYM, OVRY, SPLN, CRVX, BONE	BLD	ESC, ESDR, ESDR, BLD, LIV, MUS, VAS		Pbx-1
5	112862318	0.95	rs372492	A	G	0.81	intronic	ESC, ESDR, LNG, IPSC, FAT, STRM, BRST, BLD, BRN, SKIN, LIV, GI, ADRL, KID, PANC, MUS, PLCNT, THYM, HRT, CRVX, VAS, BONE	BLD, VAS, BRN, HRT, OVRY, PLCNT, GI, SPLN	ESDR, BLD, LIV, MUS, VAS		AP-1, E2F, Irf, NF-Y, OTX, Pbx3, RFX5, SP2
5	112864277	0.94	rs351769	C	T	0.81	intronic	BRST	FAT, STRM, BLD, MUS, BRN, SKIN, LIV, CRVX, VAS, LNG, BONE			Pax-2, Pax-8, Pou3f3, STAT
5	112865587	0.94	rs463229	G	A	0.81	intronic					Irf, Mef2, Mrg, PTF1-beta, Tgif1
5	112865587	0.86	rs80092672	G	T	0.8	intronic					BDP1
5	112869510	0.95	rs439456	G	C	0.81	intronic		ESDR, BLD			Nr2f2
5	112871119	0.94	rs460301	G	A	0.81	intronic		FAT, BLD	SKIN, MUS, BLD	CEBPB	Evi-1, Homez, Sox

Table S4. *Cont.*

Chromosome	Position	LD (r^2)	SNP ID	Reference Allele	Alternate Allele	ASN Frequency	Variant Type	Promoter Histone Marks	Enhancer Histone Marks	DNAse	Proteins Bound	Motifs Changed
5	112871310	0.95	rs2909904	G	C	0.81	intronic		FAT, BLD		CEBPB	
5	112874647	0.95	rs382260	C	T	0.81	intronic		BLD			
5	112876089	0.95	rs712671	T	C	0.81	intronic		ESC, FAT, BLD	SKIN, SKIN, PLCNT, CRVX, BRST, MUS	CEBPB	AP-1
5	112876911	0.95	rs9011	G	A, T	0.81	3'-UTR		ESC, IPSC, BLD			
5	112877374	0.95	rs2545167	G	T	0.81	3'-UTR		ESC, IPSC, BLD, SKIN			Arid3a, Pax-2, Pou3f1
5	112878108	0.95	rs2545166	C	G	0.81	3'-UTR		ESC, ESDR, BRST, BLD, SKIN, HRT, BRN		BCL3	
5	112880692	0.93	rs419632	C	T	0.19	intronic		BLD, MUS	MUS, MUS		p300
5	112882040	0.93	rs35726351	G	A	0.19	intronic					Gfi1, Pitx2
5	112883003	0.93	rs1093677	T	C	0.19	intronic			PLCNT		Pax-5
5	112884859	0.93	rs864682	C	T	0.19	intronic		HRT			Cdx2, Hoxa10, Hoxa9, PRDM1
5	112886997	0.92	rs712662	C	G	0.19	intronic	BLD	BLD, BRN, GI, HRT, LNG	BLD		GR, Rad21, TATA, THAP1, YY1
5	112887904	0.92	rs712664	A	C	0.19	intronic		BLD			Nkx2, Pax-6, Znf143
5	112888172	0.93	rs66741904	AG	A, AAAA	0.19	intronic		BLD			
5	112888634	0.89	rs458451	T	C	0.2	intronic		BLD			Foxj2, Smad
5	112888692	0.89	rs455412	G	T	0.2	intronic		BLD			Arid3a, Cart1, Dbx1, Esx1, HNF1, Hlx1, Hoxa3, Hoxa5, Hoxb4, Hoxb8, Hoxc6, Hoxd8, Lhx3, Ncx, Pax7, Pou2f2, Pou3f1, Pou3f2, Prrx2, Sox, Zfp105
5	112889255	0.87	rs456313	G	C	0.19	intronic		BLD			FAC1, Hand1, LUN-1, Pax-4, RREB-1, Smad3

Table S4. *Cont.*

Chromosome	Position	LD (r^2)	SNP ID	Reference Allele	Alternate Allele	ASN Frequency	Variant Type	Promoter Histone Marks	Enhancer Histone Marks	DNAse	Proteins Bound	Motifs Changed
5	112889451	0.92	rs460832	G	A	0.19	intronic		BLD			Fox, Foxa, Foxi1, Foxj2, Pou1f1, Pou5f1
5	112890495	0.84	rs818426	G	A	0.21	intronic		BLD			Nkx2, Rad21, SP1, ZBTB7A
5	112890958	0.89	rs818425	C	T	0.2	intronic					AP-1, BATF, GATA, HMGN3, Irf, KAP1, LXR, Maf, Nkx2, RXRA
5	112891177	0.89	rs818424	G	A	0.2	intronic					GR, HNF4, VDR
5	112891280	0.93	rs818423	C	T	0.19	intronic					GR
5	112892102	0.93	rs712665	A	G	0.19	3'-UTR					
5	112892242	0.9	rs712666	T	C	0.19	3'-UTR			PLCNT, MUS		CTCF, Pax-4
5	112892970	0.93	rs430665	G	A	0.19	3'-UTR					AP-1, BHLHE40, CTCF
5	112893472	0.84	rs466443	T	C	0.21	intronic					AP-1, BRCA1, NF-Y, Pbx3, SP2
5	112893826	0.92	rs434090	A	G	0.19	intronic		BRN, HRT			CTCF
5	112893991	0.92	rs519397	T	C	0.19	intronic		BRN, HRT			BATF, Barhl1, Cdx2, Hoxa10
5	112894128	0.82	rs200434062	T	TG	0.18	intronic		HRT			FAC1, Foxa, Foxj1, Foxk1, Foxo, Foxp1, HDAC2, Irf, Nanog, Pax-4, RREB-1, Sox, Zfp105, p300
5	112894206	0.88	rs461424	G	A	0.2	intronic		HRT			BCL, Ets, GLI, Irf, SP1, Znf143
5	112894634	0.92	rs465454	T	C	0.19	intronic		HRT			Barx1, DMRT3, Sox
5	112895753	0.92	rs461866	G	A	0.19	intronic	HRT	BRN, FAT, GI, MUS, PANC	HRT		DMRT3
5	112896132	0.92	rs469663	A	G	0.19	intronic		BRN, FAT, LIV, GI, HRT, PANC, MUS, BLD		PU1	AIRE, DMRT1
5	112896531	0.92	rs455469	A	T	0.19	intronic		SKIN, LIV, BRN, HRT, PANC, BLD		BATF, MEF2C	Foxp1, GATA, HDAC2, Irf, p300
5	112898801	0.92	rs151978	T	G	0.19	intronic					Hoxa9, TATA
5	112898852	0.92	rs151977	A	T	0.19	intronic					CEBPB, CEBPG, Hdx, Mef2, Pax-4

Table S4. *Cont.*

Chromosome	Position	LD (r^2)	SNP ID	Reference Allele	Alternate Allele	ASN Frequency	Variant Type	Promoter Histone Marks	Enhancer Histone Marks	DNAse	Proteins Bound	Motifs Changed
5	112900751	0.92	rs151976	C	T	0.19	intronic		HRT			ZBTB7A
5	112903185	0.92	rs153545	T	C	0.19	intronic					Dbx1, Gfi1, Hoxd10, Mef2, Pax-4, Pou3f2, Sox
5	112905555	0.84	rs200722994	AC	A	0.19	intronic					DMRT7, FAC1, Foxa, Foxj1, Foxk1, Foxo, Foxp1, HDAC2, Irf, Nanog, PTF1-beta, Pax-4, RREB-1, Sox, Zfp105, p300
5	112905563	0.8	rs818782	A	C	0.18	intronic					DMRT7, FAC1, Fox, Foxa, Foxd3, Foxf1, Foxi1, Foxj1, Foxj2, Foxk1, Foxl1, Foxo, Foxp1, Foxq1, HDAC2, Irf, Pax-4, RREB-1, Sox, Zec, Zfp105, p300
5	112907271	0.89	rs469727	C	T	0.18	intronic	MUS	BRST, BLD, FAT, BRN, GI, MUS, HRT, OVRY, PANC, SPLN	HRT, MUS, MUS, MUS, SKIN		
5	112907830	0.89	rs698405	C	T	0.18	intronic		BRN, MUS			GATA, RXRA
5	112908726	0.89	rs446567	T	C	0.18	intronic					Foxp1, HDAC2, Pax-4, Pou6f1, SIX5
5	112909080	0.88	rs469760	G	A	0.18	intronic		OVRY, HRT	SKIN		
5	112910273	0.89	rs468783	G	T	0.18	intronic		ESDR, BRN			
5	112914501	0.85	rs193527	C	T	0.18	intronic		LNG, BLD, SKIN, ADRL, HRT, GI, MUS, CRVX, BONE			EWSR1-FLI1, GR, Ik-1, ZBTB7A

The risk SNP identified in this study is in boldface.
